# Total DNA Methylation Changes Reflect Random Oxidative DNA Damage in Gliomas

**DOI:** 10.3390/cells8091065

**Published:** 2019-09-11

**Authors:** Anna-Maria Barciszewska, Małgorzata Giel-Pietraszuk, Patrick M. Perrigue, Mirosława Naskręt-Barciszewska

**Affiliations:** 1 Intraoperative Imaging Unit, Chair and Clinic of Neurosurgery and Neurotraumatology, Karol Marcinkowski University of Medical Sciences, Przybyszewskiego 49, 60-355 Poznan, Poland; 2 Department of Neurosurgery and Neurotraumatology, Heliodor Swiecicki Clinical Hospital, Przybyszewskiego 49, 60-355 Poznan, Poland; 3 Institute of Bioorganic Chemistry, Polish Academy of Sciences, Noskowskiego 12/14, 61-704 Poznan, Poland

**Keywords:** 8-oxo-deoxyguanosine, 5-methylcytosine, glioma, biomarker, oxidative damage

## Abstract

DNA modifications can be used to monitor pathological processes. We have previously shown that estimating the amount of the main DNA epigenetic mark, 5-methylcytosine (m^5^C), is an efficient and reliable way to diagnose brain tumors, hypertension, and other diseases. Abnormal increases of reactive oxygen species (ROS) are a driving factor for mutations that lead to changes in m^5^C levels and cancer evolution. 8-oxo-deoxyguanosine (8-oxo-dG) is a specific marker of ROS-driven DNA-damage, and its accumulation makes m^5^C a hotspot for mutations. It is unknown how m^5^C and 8-oxo-dG correlate with the malignancy of gliomas. We analyzed the total contents of m^5^C and 8-oxo-dG in DNA from tumor tissue and peripheral blood samples from brain glioma patients. We found an opposite relationship in the amounts of m^5^C and 8-oxo-dG, which correlated with glioma grade in the way that low level of m^5^C and high level of 8-oxo-dG indicated increased glioma malignancy grade. Our results could be directly applied to patient monitoring and treatment protocols for gliomas, as well as bolster previous findings, suggesting that spontaneously generated ROS react with m^5^C. Because of the similar mechanisms of m^5^C and guanosine oxidation, we concluded that 8-oxo-dG could also predict glioma malignancy grade and global DNA demethylation in cancer cells.

## 1. Introduction

Gliomas are the largest and most diverse group of primary brain tumors [1]. They are subdivided according to their histological and molecular features [2]. Diffuse gliomas, the most abundant subgroup, show broad infiltration in the surrounding central nervous system (CNS) parenchyma. They present the malignancy range from WHO grade II (low grade) to IV (glioblastoma, high grade). Other glial tumors, like pilocytic astrocytomas (WHO grade I), have a more circumscribed growth pattern. Glioblastoma (GBM) is the most frequent diffuse glioma in adults and is the deadliest form with a median patient survival of about one year. GBM cells have the predilection for aggressive invasion into the normal brain tissue and infiltration along the vascular tracts, which limits the possibility of complete tumor resection and reduces the effect of localized radiotherapy. Defects in multiple intracellular signaling pathways are involved in the gliomas’ pathogenesis and lead to the evasion of cell death, which constitutes a hallmark of cancer [3].

Cellular damage accelerates the advancement of degenerative processes, aging, apoptosis, as well as carcinogenesis [4,5]. Reactive oxygen species (ROS) are the main damaging agent of various cell components, including DNA [4]. Under physiological conditions, there is a balance between ROS production and scavenging, as well as oxidative damage to cellular components and their repair [6]. An imbalance between ROS production, antioxidant enzymes, and radical scavenging compounds’ activities indicates cellular stress [7]. Excessive ROS levels are pro-tumorigenic and lead to the activation of pro-survival signaling pathways, generation of oncogenic mutations, loss of tumor suppressor gene-function, adaptations to hypoxia, and increased glucose metabolism [8,9]. Therefore, quantification of ROS in cells has great informative potential for medical diagnosis.

Direct measurement of ROS in biological material is possible, but difficult to apply in the clinical practice [10]. More reliable and useful information is harbored in cellular components, such as the amount of nucleic acid already oxidized by ROS, which can be determined [11,12].

Hydroxyl radical (•OH) is the most reactive among ROS and has a relatively short half-life (10^−9^ s–10^−8^ s). It generates a vast range of DNA lesions, including canonical and odd bases modifications, deletions, strand breakages, and chromosomal rearrangements. DNA damage products, being a result of •OH activity, can be used as indicators of cellular oxidative stress. The product of a direct reaction of •OH with guanosine, the most easily oxidizable base, is 8-oxo-7,8-dihydro-2′-deoxyguanosine (8-oxo-dG). 8-oxo-dG mispairs also with adenine, instead of cytosine, and induces G→T transversions [13]. Therefore, oxidative stress and, in consequence, a higher amount of 8-oxo-dG in DNA contribute to changes in cancer cells, such as elevated proliferation rate, chemotherapy resistance, and metastatic ability.

Oxidative damage can also occur with modified DNA constituents, including 5-methylcytosine (m^5^C), which is the main epigenetic marker [14]. m^5^C is most commonly found in CpG islands, which are short stretches of DNA with the CG sequence frequency higher than in other regions. m^5^C presence in DNA influences cell differentiation and development processes [15,16]. The first and most significant epigenetic aberration found in cancer cells is abnormal DNA methylation patterns in CpG islands. In certain cancers, m^5^C becomes continuously eroded throughout the genome. However, some promoter-associated CpG islands remain or become hypermethylated to act in a process known as ‘CpG island methylator phenotype’, which essentially inactivates tumor suppressor genes [17]. Therefore, DNA methylation changes (m^5^C) are regarded as a good measure of cancer development and progression [18]. We have previously shown that the total contents of m^5^C can serve as a marker in the diagnosis of various diseases and aging, as well as for the molecular description of the treatment process [19,20,21,22,23]. Therefore, the implementation of total DNA methylation analysis, rather than more specific methods (like methylation matrices, methylome sequencing), is a useful approach in clinical practice [24,25].

Our study aimed to provide an insight into the potential mechanism and effects of m^5^C demethylation and oxidative DNA damage in m^5^CpG dinucleotides of cancer cells. We investigated the relation of m^5^C and 8-oxo-dG amounts, estimated in DNA isolated from brain glioma tissues and peripheral blood samples, to tumor grade. Our findings revealed a correlation in the level of m^5^C and 8-oxo-dG that corresponds to glioma malignancy. We noticed an increased level of oxidized guanosine (8-oxo-dG) and loss of m^5^C (demethylation) in DNA from brain glioma tissues and peripheral blood samples. Our data suggested that dynamic demethylation of m^5^C in cancer cells was due to random hydroxyl radicals reacting with DNA. To explain that, we described in detail the similarities in mechanisms of m^5^C loss and guanosine oxidation product formation. We proposed 8-oxo-dG as a solid auxiliary marker for grading gliomas. That additionally reinforced the m^5^C position as a marker of cancer development and progression through DNA damage and epigenetic deregulation.

## 2. Materials and Methods

### 2.1. Collection of Tumor Tissue and Peripheral Blood Samples

The brain glioma tissue samples for this study were collected from 28 glioma patients who underwent tumor surgery at the Department of Neurosurgery and Neurotraumatology of the University of Medical Sciences in Poznan between 2016 and 2017. Peripheral whole blood samples (4.9 mL, in EDTA-coated vials) from another 8 glioma patients were taken preoperatively. The control group consisted of 34 generally healthy individuals, from whom peripheral blood samples were taken. The samples were stored frozen at −80 °C. Demographic and clinical data were collected from the patients’ medical records. Tumors were graded histopathologically, according to the WHO classification.

Peripheral blood and tumor tissue analyses were approved by the Bioethical Committee of Karol Marcinkowski University of Medical Sciences, Poznan (896/9, 838/12). The participants provided a written consent to donate their tissue and blood samples for research.

### 2.2. Isolation of DNA from Tumor Tissue Samples and Peripheral Blood Samples

DNA isolation from blood and tissue samples was performed, as previously described [19,20,21,23]. DNA purity was controlled with UV absorbance measurements at 260 and 280 nm. In the analyzed set, the A_260_/A_280_ ratio for all samples was 2.0–2.1.

### 2.3. Analysis of m^5^C Contents in DNA

DNA hydrolysis, labeling, and TLC chromatography were performed as previously described [19,20,21,22,23]. The radioactive intensity of spots corresponding to m^5^C, C (cytosine), and T (thymine) was used for calculations. The data obtained with the post labeling and TLC methods were compared to the ELISA assay [26].

### 2.4. Analysis of 8-oxo-dG Contents in DNA

DNA was dissolved in 200 µL of a buffer (pH 5.3) containing 40 mM sodium acetate and 0.1 mM ZnCl2, then mixed with nuclease P1 (Sigma-Aldrich, St. Louis, MO, USA) solution (30 µg), and incubated for 3 h at 37 °C. Then, 30 µL of 1M Tris–HCl pH 8.0 and 5 µL of alkaline phosphatase (1.5 units) solution were added, following with 1 h incubation at 37 °C. DNA hydrolysate was purified using cut-off 10,000 Da filter units. 8-oxo-dG amount in DNA was determined using HPLC (Agilent Technologies 1260 Infinity, Santa. 25. Clara, CA, USA) with two detectors working in series: 1260 Diode Array Detector and Coulochem III Electrochemical Detector (ESA Inc., Chelmsford, MA, USA). Isocratic chromatography of DNA hydrolysate was performed using a solution of 50 mM CH_3_COONH_4_ at pH 5.3 and methanol (93:7). Analysis of dG for reference was performed at 260 nm. 8-oxo-dG was determined with the following electrochemical detection settings: guard cell +400 mV, detector 1: +130 mV (screening electrode), detector 2: +350 mV (measuring electrode set on the 100 nA sensitivity).

### 2.5. Calculation of the Total Amount of M^5^c And 8-Oxo-Dg in Human DNA

The amount of modified bases in DNA was calculated on the basis of global genome composition—3.2 × 10^9^ bases (100%), where C—663 × 10^6^ (20%), T—954 × 10^6^ (29%), G—663 × 10^6^ (20%), A—954 × 10^6^ (29%), and m^5^C—33 × 10^6^ (1%) [27]. Amount of m^5^C (%) in pyrimidines in DNA was determined from TLC analysis with formula R[%] = m^5^C × 100/(C + T). The total number of m^5^C in the genome was calculated from formula m^5^C = (1,584,257,992) × R/100 [27].

**Figure 1:**
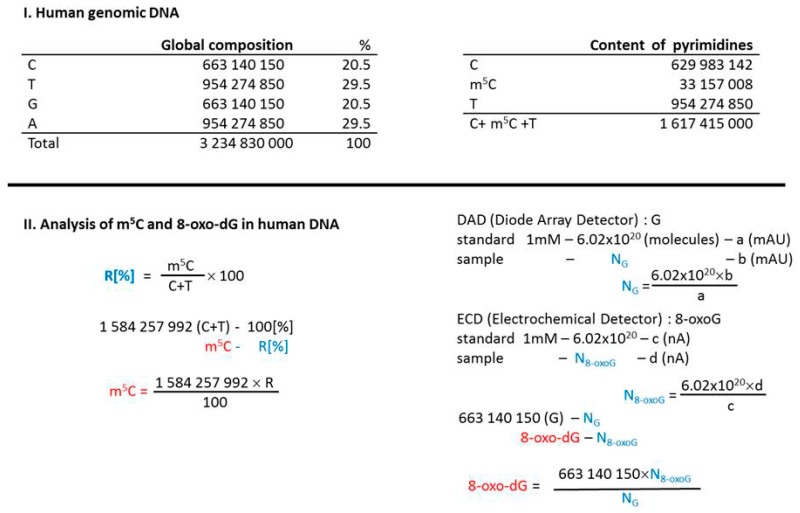
Calculations leading to determine the 5-methylcytosine (m^5^C) and 8-oxo-deoxyguanosine (8-oxo-dG) contents in examined DNA samples. Human genomic DNA composition data were taken from [27].

The input amount of guanosine was necessary to determine 8-oxo-dG contents. It was calculated from Diode Array Detector (PAD) measurements using Avogadro number N_G_ = 6.02 × 10^20^ × b(mAU)/a(mAU) standard. 8-oxo-dG nucleoside amount was estimated with electrochemical detector N_8-oxo-dG_ = 6.02 × 10^20^ × d(nA)/c(nA) standard. The total number of 8-oxo-dG = 663 × 10^6^ × N_8-oxo-dG_/N_G_ (Figure 1).

### 2.6. Statistical Analysis

Unless otherwise specified, the results were expressed as the mean ± SD (Standard Deviation) in the error bars, and the data were collected from three independent analyses. Statistical significance was determined using the STATISTICA software, 1998 edition (StatSoft Poland, 1995–2008), as in our previous studies [28], by ANOVA test, and a *p*-value <0.05 was considered as significant.

## 3. Results

### 3.1. Patients’ Characteristics

The analyzed cohort consisted of 36 individuals diagnosed with brain glioma, aged from 19 to 81 years (Table S1 and S2). The median patient’s age at the time of tumor surgery was 53.1 ± 13.3 years. There were 21 (58.3%) males and 15 (41.7%) females. The tumors were mainly primary (i.e., the samples were taken before any other oncological treatment), but the cohort included some cases of recurrent gliomas (those undergone previously first surgery, radiotherapy, and chemotherapy) (Table S1 and S2). The control group consisted of 34 generally healthy persons, aged from 18 to 66 years (mean 43.1 ± 14.5 years), with 15 (44.1%) males and 19 (55.9%) females (Table S3).

### 3.2. Total Contents of m^5^C and 8-oxo-dG in DNA from Tumor Tissue Samples

**Figure 2:**
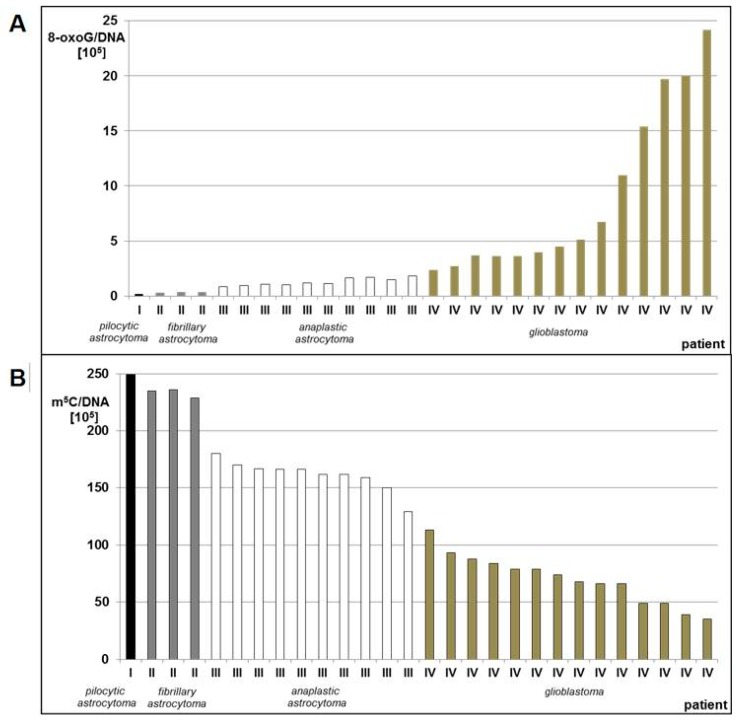
8-oxo-dG (**A**) and m^5^C (**B**) contents in DNA from human brain tumors tissues classified as pilocytic astrocytoma (WHO I), fibrillary astrocytoma (WHO II), anaplastic astrocytoma (WHO III), and glioblastoma (WHO IV).

For 28 patients, the total level of m^5^C and 8-oxo-dG in genomic DNA from brain tumor tissue was analyzed (Table S1). There were variations in the contents of both compounds in the tumor histological subgroups, most clearly seen for glioblastoma group, reflecting the heterogeneity of those tumors (Table S1, Figure 2).

We observed a significant stepwise increase of 8-oxo-dG contents in DNA from brain glioma tissue with increasing tumor malignancy. Glioblastoma (WHO IV) had 12–121 times higher amount of 8-oxo-dG than pilocytic astrocytoma (WHO I) (Table S1, Figure 2A). Low levels of total DNA methylation were concomitant with high levels of 8-oxo-dG and in higher grade gliomas. The m^5^C amount in pilocytic astrocytoma (WHO I) was 2–7 times higher than glioblastoma (WHO IV) (Table S1, Figure 2B). The number of m^5^C residues in relation to the number of 8-oxo-dG residues was ca. 1000:1 in low grade (WHO I-II) gliomas, and dropped significantly to ca. 100:1 or less in high grade (WHO III-IV) tumors, indicating higher DNA damage (Table S2).

**Figure 3:**
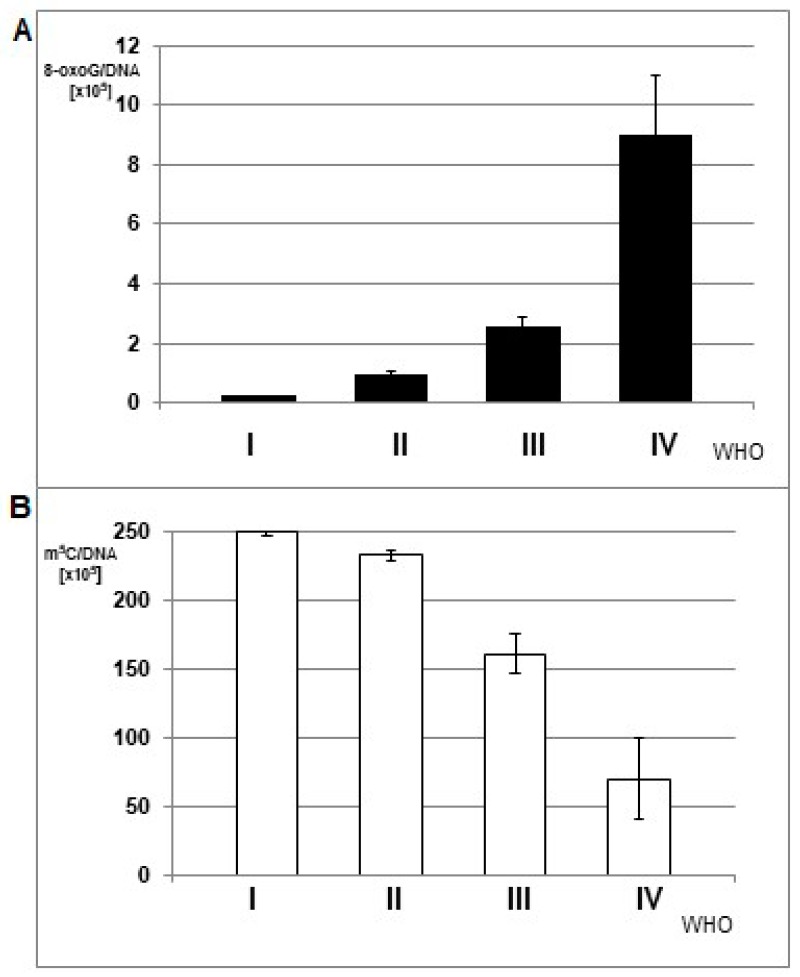
The mean amount of 8-oxo-dG (**A**) and m^5^C (**B**) in DNA from brain glioma tissues of different malignancy grade (WHO I-IV).

The mean amounts of 8-oxo-dG were increasing with increasing tumor grade, while total DNA methylation was decreasing (Figure 3). The differences between the subgroups comprising tumors of the same grade were statistically significant. The ANOVA test for 8-oxo-dG showed F= 4.873 and *p* = 0.009; only for WHO groups III and IV, F = 10.346, *p* = 0.004; whereas for m^5^C, F = 107.194 and *p* < 0.005.

**Figure 4:**
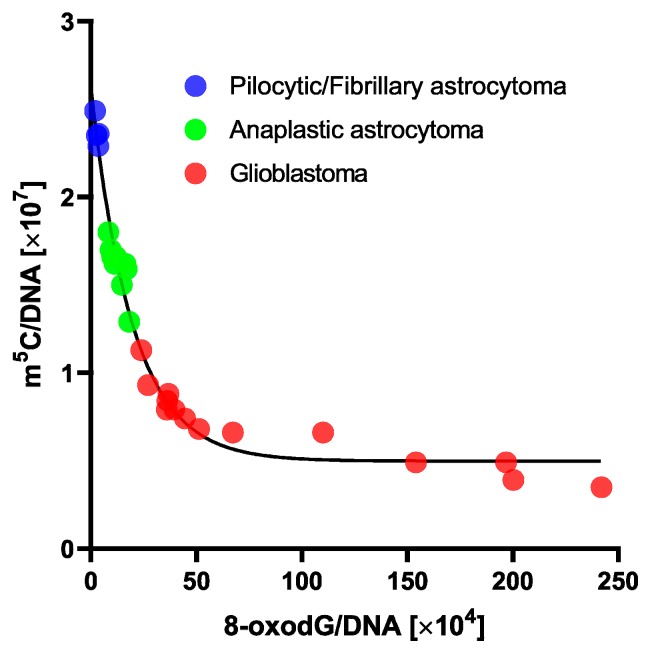
One phase decay curve of m^5^C in relation to 8-oxo-dG contents in DNA from human brain tumors tissues. 8-oxo-dG allows subdividing gliomas within a similar total DNA methylation level.

The application of an exponential one-phase decay function for m^5^C in relation to 8-oxo-dG showed an extremely high correlation coefficient of 0.9804 (Figure 4). A clear trend was observed that a small increase in 8-oxo-dG contents led to great depletion in m^5^C amount.

### 3.3. Total DNA Methylation and 8-oxo-dG Contents in Peripheral Blood Samples

**Figure 5:**
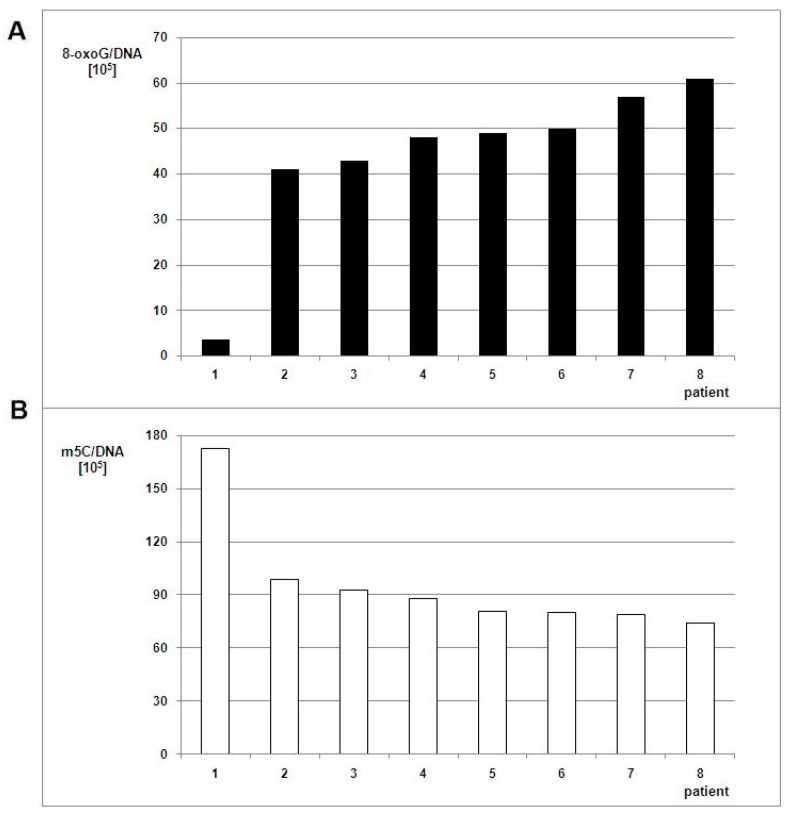
8-oxo-dG (**A**) and m^5^C (**B**) contents in DNA from the peripheral blood of patients with brain tumors. The white bars correspond to m^5^C contents, the black ones to the number of 8-oxo-dG nucleosides in DNA.

For eight glioma patients, we analyzed the total level of m^5^C and 8-oxo-dG in genomic DNA from peripheral blood samples (Table S2). The group included seven glioblastoma (WHO IV) patients along with one anaplastic astrocytoma (WHO III) for comparison. The total DNA methylation levels were almost equal in that group (Table S2, Figure 5).

The relationship of m^5^C and 8-oxo-dG contents observed for peripheral blood samples was similar to those from glioma tissues. The amount of m^5^C was 1.7–2.3 times lower and 8-oxo-dG 11.4–16.9 times higher in WHO IV samples when compared to the WHO III sample.

**Figure 6:**
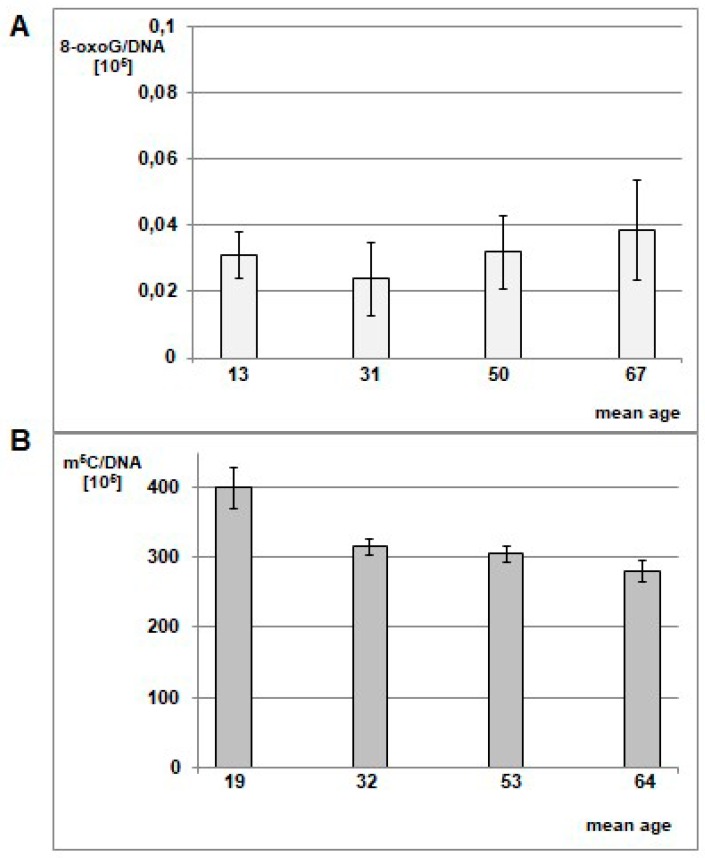
Mean level of 8-oxo-dG (**A**) in leukocyte DNA in healthy individuals cohort divided into mean age groups of 13, 31, 50, and 67 y.o. The graph was prepared based on data taken from Siomek et al. 2007 [30], 8-oxo-dG values were recalculated per genome. One can see the minute amounts of 8-oxo-dG in healthy subjects. (**B**) Mean amount of m^5^C in DNA from peripheral blood samples of healthy individuals in different age groups. The cohort (Table S3) was divided into 4 age groups: <21 y.o. (mean age 19), 21–40 y.o. (mean age 32), 41–60 y.o. (mean age 53), >60 y.o. (mean age 64). The amounts of m^5^C were significantly higher than in glioma patients.

The differences between m^5^C and 8-oxo-dG contents for blood samples in glioma patients versus the control group were statistically significant. In the control group of generally healthy individuals, the mean m^5^C contents in DNA was 314 ± 31 × 10^5^ (Figure 6), so significantly higher than in the brain glioma WHO III-IV group (Table S2, Figure 5). It has been already reported that the amount of 8-oxo-dG in normal human cells’ DNA is of 1 base per 10^7^ guanosine residues [29]. The relation of the 8-oxo-dG contents with subjects’ older age showed clear increase: 4.63 ± 1.11 (for mean age 13), 3.59 ± 1.60 (for mean age 31), 4.82 ± 1.62 (for mean age 50), and 5.79 ± 2.22 (for mean age 67) per 10^6^ guanosine residues [30]. We calculated 8-oxo-dG amounts per whole genome (DNA). The 8-oxo-dG contents in peripheral blood DNA of glioma patients were substantially higher. The one-way ANOVA test values were F = 309.347 and *p* < 0.005 for total DNA methylation in glioma and control group, and F = 49.966 and *p* < 0.005 for 8-oxo-dG contents between glioma group and the literature data [29].

## 4. Discussion

Currently, there is a need for better complex disease characterization on the molecular level that can assist clinicians in identification and optimal treatment of brain gliomas. Molecular markers turned out to be a powerful aid for clinical diagnostics, precise treatment implementation, and estimating clinical outcomes [31]. DNA hypomethylation is an exceptionally frequent change linked to numerous neoplasms [32,33]. Carcinogenesis is also linked to the overproduction of reactive oxygen species, including superoxide radical, hydroxyl radical and hydrogen peroxide, as well as a shortage of antioxidants [34]. ROS levels in cancer cells are typically higher than physiologic because of increased metabolic activity, cellular signaling, oncogene activity, and mitochondrial dysfunction [35,36].

The brain is especially susceptible to the damaging effects of ROS because of its high metabolic activity, oxygen consumption, and low capacity for cellular regeneration [5]. The impact of ROS, as well as reactive nitrogen species (RNS), on gliomagenesis have been described, mainly in signal transduction and metabolomics aspects [37,38].

We previously showed that there’s a clear correlation between total DNA methylation and brain tumor malignancy, in tumor tissues as well as in peripheral blood samples [19,21,23]. We also identified single papers that estimated 8-oxo-dG in glioma tissues, noticing the relation of higher oxidative stress with tumor malignancy [39,40,41]. In the present study, we focused on the comparison between m^5^C, a DNA epigenetic marker, and 8-oxo-dG, which is an established well-known oxidative damage marker, in glioma tissues and peripheral blood samples. Both nucleosides frequently occur in m^5^CpG dinucleotides [42]. One previous study on genome-wide methylation profiling in pediatric glioblastoma showed a possible association of ROS with pediatric GBM. Gene Ontology analysis performed there showed that the pediatric GBM-specific methylome identified the “superoxide metabolic process” and the “oxygen and ROS metabolic process”, indicating ROS action as a driving phenomenon in those tumors [43]. What is unique in our present findings is that for the first time we’ve shown a direct correlation between the oxidative DNA damage (monitored by 8-oxo-dG) and epigenetic regulation (through total m^5^C), and their relation with tumor malignancy. Moreover, 8-oxo-dG allowed subdividing gliomas within a similar total DNA methylation level (Figure 4). That is a step further than nowadays existing classification systems [1], and holds a potential for better patient stratification in terms of treatment planning and prognosis.

The •OH radical diffusion rate properties and ability to react with DNA is confined to short distances, limited to 2 nm in cells and tissues. Therefore, it acts seemingly at generation sites [44]. The Fenton reaction is the most common source of •OH in the cell [45]. It involves reduced redox-active metal ions, as ferrous and cuprous, that react with metabolically produced hydrogen peroxide (H_2_O_2_). There is usually the site-specific output of •OH from Fenton-like reactions, for example, including metal ions close to or bound to DNA [44].

Guanine (G) is the most susceptible to oxidation (lowest redox potential, 1.29 mV) out of the four DNA bases [42]. The 8-oxo-dG redox potential is even lower, 0.74 mV, which results in its further oxidation [46]. That property is explored for electrochemical analyses of this compound at a picomol level [47]. To determine the extent of oxidative stress to which a cell has been exposed, the cellular level of 8-oxo-dG in the genome can be estimated and regarded as a status of oxidative stress leading to mutations [48]. The frequency for 8-oxo-dG to cause de novo spontaneous and heritable G→T transversion mutations in mice was estimated at the range of ~1% [49]. There is a steady-state level of ~30,000 8-oxo-dGs (mouse embryonic stem cell) that theoretically generates ~300 mutation events during replication [50]. m^5^C deamination can also lead to C→T transition [51]. In mouse embryonic stem cells there are ~50 million m^5^C sites that could result in ~1000 mutations [50]. Therefore, it can be estimated that m^5^C leads to ~3-fold more mutations than 8-oxo-dG. That corresponds to deep genome sequencing studies that identified more C→T than G→T mutations [52]. Moreover, the presence of 8-oxo-dG negatively affects adjacent sites of DNA methylation and suppresses methylation of nascent DNA strand one or two base pairs away from the damaged guanine [53,54]. On the other hand, DNA methylation may influence the mutagenic effect of ROS [55].

The demarcation between genetic (mutations) and epigenetic DNA modifications is blurred. The oxidative damage to m^5^C is extremely important for the formation of epimutations [56], as well as for its function as an epigenetic regulator [15,16,32]. Therefore, it should lead to a reevaluation of modifications that are considered mutagenic.

A parallel analysis of m^5^C (epigenetic mark) and 8-oxo-dG (oxidation marker) provides a challenge of the biological significance of an enzymatic m^5^C hydroxylation mechanism leading to DNA demethylation in brain tumors. Data are showing that Ten-eleven translocation (TET) proteins are responsible for m^5^C oxidation and, in fact, demethylation [57]. They cause hydroxylation of the m^5^C methyl group forming 5-hydroxymethylcytosine (5hmC), 5-formylcytosine (5fC), and 5-carboxylcytosine (5caC). TET-mediated m^5^C oxidation is considered as an active DNA demethylation mechanism for genome stability. DNA demethylation sites are concomitant with base excision repair. 8-Oxoguanine glycosylase (OGG1) binds and recruits TET1 to the 8-oxo-G adjacent m^5^C sites to prevent mutations [58]. Tet2 is responsible for marking sites of DNA-damage with 5hmC [59]. Cancer genomes are characterized by focal DNA hypermethylation, co-existing with widespread hypomethylation. However, recent data show, that even in the case of TET loss of function, general loss of DNA methylation occurs [60]. That raises the question about the exact mechanism of oxidative DNA modifications.

**Figure 7:**
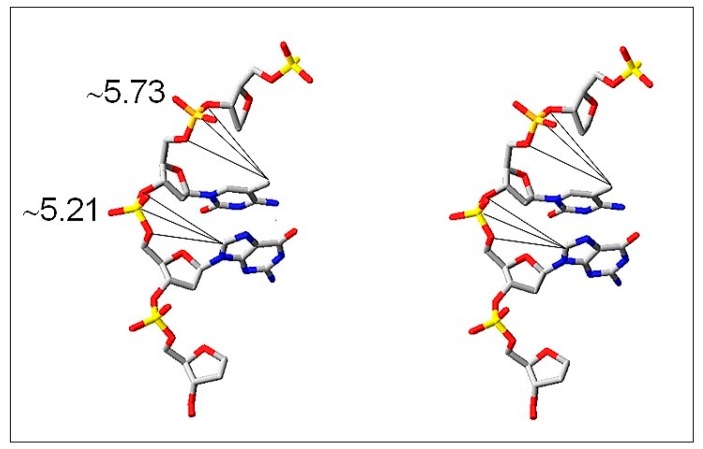
Stereo view of the major groove of DNA where m^5^C and guanosine (C-8) are located and are freely available for the reaction with •OH. It is indicated by distances (Å) of the reactive sites (-CH_3_ of m^5^C and C-8 of G) from a phosphodiester group of the nucleotide (modified from [61]).

It is generally accepted that 8-oxo-dG formation is the result of the attack of •OH on carbon 8 (C8) of guanosine, which is the most reactive site easily available to •OH in the major groove of DNA (Figure 7).

The question arises whether m^5^C, a neighbor nucleoside in m^5^CpG dinucleotide, is reacting with •OH (Figure 7). The hydrophobic methyl group of m^5^C protrudes into the major groove of DNA double helix and is prone to •OH attack. A direct oxidation product of m^5^C is 5hmC [62]. Moreover, oxidation of m^5^C induces final loss of a methyl group in the form of formaldehyde or formic acid [63]. Further support for that observation is a relatively low ratio (3–5) of the methyl group modification in comparison to guanosine modification. It is a result of a small efficiency of hydrogen abstraction from a methyl group and formation of C–CH_2_•. Our data provide strong support for ROS-induced demethylation of DNA in glioma patients’ samples. Furthermore, it was demonstrated that ROS-mediated DNA oxidation that yields 8-oxo-dG in gene promoters is a signal for gene activation. The presence of 8-oxo-dG in a G-quadruplex-forming promoter sequence resulted in ca. 300% in gene expression increase [64]. A comparison of 8-oxo-dG yield and m^5^C loss in human brain tumors of different malignancy strongly suggests that both nucleosides are modified by ROS, particularly by •OH, during oxidative stress conditions.

There is no doubt that elevated ROS rates promote tumor development and progression [36]. However, the relations of DNA methylation changes with cancer are less obvious. Cancer-linked DNA hypermethylation includes promoters of tumor suppressor genes resulting in repression of gene expression, thereby facilitating cancer formation. Global DNA hypomethylation during the neoplastic process doesn’t have such straightforward relation but is observed in many tumors, and results in global gene expression deregulation [65]. It is also observed during cellular senescence [20]. It is generally accepted that the antineoplastic effect of chemotherapeutic drugs is due to the induction of oxidative stress and ROS-mediated cell injury [66]. In the same way, DNA demethylation agents are used [67]. But is the way of complete oxidative damage of the cell the right way of oncological treatment? The cells that survive such therapy are highly resistant to any treatment and produce aggressive recurrences. Moreover, significant harm is done to normal organism’s cells [66]. Based on the results of our study, it may be presumed that the opposite actions should be promoted and DNA hypermethylating strategies should be concerned. Antioxidants’ intake is a well-known prophylactic and treatment supporting method. They generally increase the response rate to chemotherapy, help restore proper redox balance of the organism, and some even impact patients’ survival [68]. The examples are also known for brain gliomas [69]. Besides adding them to the therapeutic regimen, one can explore the ROS-scavenging properties of already used drugs. In the case of brain gliomas, mannitol, the widely used medication against brain edema, presents such effects [70]. That detail can impact the extension of its use in therapy.

## 5. Conclusions

We showed that brain gliomas were characterized by strong oxidative stress, which resulted in global DNA damage. Two different types of DNA modifications, mainly occurring randomly, were characterized. 8-oxo-dG levels were indicative for increased oxidative stress, DNA damage, and therefore higher tumor malignancy. In parallel, DNA demethylation was observed, resulting in pathological DNA gene expression.

## Supplementary Materials

The following are available online at https://www.mdpi.com/2073-4409/8/9/1065/s1. Table S1. The characteristics of 28 patients for whom the contents of m^5^C (post labeling method) and 8-oxo-dG (electrochemical detector) in DNA from tumor tissue were analyzed, as well as patients’ clinical characteristics. Table S2. The characteristics of 8 patients with WHO III and IV brain gliomas for whom the contents of m^5^C (post labeling method) and 8-oxo-dG (electrochemical detector) in DNA from peripheral blood samples were analyzed, as well as patients’ clinical characteristics. Table S3. The characteristics of 34 healthy individuals comprising the control group for whom the contents of m^5^C (postlabeling method) in DNA from peripheral blood samples was analysed.
