# Supplementary material for: Total DNA Methylation Changes Reflect Random Oxidative DNA Damage in Gliomas
Source: Cells. 2019 Sep 11;8(9):1065. doi: 10.3390/cells8091065 (PMC6770701; doi:10.3390/cells8091065)
Supplement: Supplementary file 1 [file cells-08-01065-s001.pdf]

Supplementary Table S1. The characteristics of 28 patients for whom the content of m^5C (postlabeling method) and 8-oxo-dG (electrochemical detector) in DNA from tumor tissue was analysed, as well as patients clinical characteristics.

**Patient**	**Glioma histological type**	**Sex**	**Age**	**WHO grade**	**8-oxodG/DNA [×10^4] ± SD**	**m^5C/DNA [×10^7] ± SD**	**m^5C/8-oxodG**	**8-oxodG/m^5C**
1	Pilocytic astrocytoma	M	66	I	1.99±0.06	2.49±0.03	1368	0.0007
2	Fibrillary astrocytoma	F	34	II	2.85±0.15	2.35±0.16	872	0.0011
3	Fibrillary astrocytoma	F	41	II	3.58±0.13	2.36±0.07	694	0.0014
4	Fibrillary astrocytoma	F	48	II	3.51±0.12	2.29±0.18	688	0.0015
5	Anaplastic astrocytoma	F	64	III	8.23±0.51	1.80±0.08	202	0.0049
6	Anaplastic astrocytoma	M	67	III	9.35±0.54	1.70±0.22	216	0.0046
7	Anaplastic astrocytoma	F	34	III	10.48±0.47	1.67±0.11	167	0.0059
8	Anaplastic astrocytoma	M	59	III	10.08±0.44	1.66±0.06	173	0.0058
9	Anaplastic astrocytoma	M	43	III	11.93±0.27	1.66±0.09	145	0.0069
10	Anaplastic astrocytoma	M	57	III	11.27±0.27	1.62±0.09	145	0.0069
11	Anaplastic astrocytoma	M	59	III	16.31±0.35	1.62±0.03	104	0.0096
12	Anaplastic astrocytoma	M	19	III	16.91±0.59	1.59±0.07	99	0.0100
13	Anaplastic astrocytoma	M	74	III	14.71±1.33	1.50±0.14	109	0.0092
14	Anaplastic astrocytoma	M	30	III	18.10±0.66	1.29±0.04	87	0.0114

15	Glioblastoma	M	59	IV	23.90±0.30	1.13±0.77	47	0.0212
16	Glioblastoma. recurrent	M	67	IV	27.12±0.75	0.93±0.26	36	0.0276
17	Glioblastoma	M	60	IV	36.75±0.25	0.88±0.06	26	0.0373
18	Glioblastoma	M	57	IV	36.21±0.75	0.84±0.05	21	0.0486
19	Glioblastoma	M	60	IV	36.01±0.42	0.79±0.08	24	0.0408
20	Glioblastoma	F	48	IV	39.63±0.78	0.79±0.07	21	0.0473
21	Glioblastoma	F	81	IV	44.56±1.15	0.74±0.14	15	0.0647
22	Glioblastoma	F	47	IV	51.30±0.20	0.68±0.12	13	0.0743
23	Glioblastoma. recurrent	M	30	IV	67.33±8.50	0.66±0.09	10	0.0985
24	Glioblastoma	F	56	IV	110.00±2.40	0.66±0.14	6	0.1611
25	Glioblastoma	F	71	IV	154.11±7.63	0.49±0.07	3	0.3018
26	Glioblastoma. recurrent	F	65	IV	196.80±0.32	0.49±0.08	3	0.3854
27	Glioblastoma	F	51	IV	200.30±3.10	0.39±0.08	2	0.5208
28	Glioblastoma	F	48	IV	242.04±8.00	0.35±0.10	1	0.6885

Supplementary Table S2. The characteristics of 8 patients with WHO III and IV brain gliomas for whom the content of m^5C (postlabeling method) and 8-oxo-dG (electrochemical detector) in DNA from peripheral blood samples was analysed, as well as patients clinical characteristics.

**Patient**	**Glioma histological type**	**Sex**	**Age**	**WHO grade**	**8-oxodG/DNA [$\times 10^5$] ± SD**	**m^5C/DNA [$\times 10^7$] ± SD**	**m^5C/8-oxodG**	**8-oxodG/m^5C**
1	Anaplastic astrocytoma	F	44	III	3.60±0.3	1.730±0.130	48.33	0.02
2	Glioblastoma	M	53	IV	41.0±0.1	0.991±0.87	2.40	0.42
3	Glioblastoma	M	46	IV	43.0±3.4	0.934±0.52	2.17	0.46
4	Glioblastoma	M	49	IV	48.0±8.4	0.877±0.131	1.83	0.54
5	Glioblastoma	M	54	IV	49.0±7.4	0.805±0.139	1.64	0.61
6	Glioblastoma	F	48	IV	50.9±2.3	0.798±0.124	1.56	0.64
7	Glioblastoma	M	62	IV	57.0±7.8	0.792±0.123	1.38	0.72
8	Glioblastoma	M	61	IV	61.0±7.6	0.740±0.095	1.22	0.82

Supplementary Table S3. The characteristics of 34 healthy individuals comprising the control group for whom the contents of m^5C (postlabeling method) in DNA from peripheral blood samples was analysed.

Patient	Sex	Age	m^5C/DNA [$\times 10^5$] ± SD
1	M	18	434.09±25.13
2	M	18	377.05±25.87
3	M	19	388.14±30.52
4	F	30	313.68±13.13
5	M	30	310.51±10.13
6	F	31	327.94±10.12
7	M	31	334.28±14.12
8	M	32	299.42±14.09
9	F	32	313.68±11.01
10	M	38	315.27±10.21
11	F	42	312.09±5.32
12	M	45	313.68±5.03
13	F	45	321.61±3.03
14	F	47	313.68±2.32

15	F	47	313.68±2.56
16	F	48	313.68±8.12
17	F	49	315.27±3.55
18	M	53	318.44±16.53
19	M	54	315.27±14.11
20	M	54	313.68±8.88
21	F	54	310.51±5.32
22	F	54	320.02±10.23
23	M	55	299.42±13.83
24	M	56	297.84±13.03
25	F	56	288.33±11.09
26	F	57	297.84±10.34
27	F	57	297.84±3.66
28	F	58	291.50±14.46
29	F	58	294.67±8.31
30	M	59	288.33±11.04
31	F	60	283.58±16.12
32	M	62	278.83±10.08

33	F	63	267.74±19.04
34	F	66	297.84±8.36
